# AgNPs–Cellulose Nanofiber/Polyacrylamide Hydrogels as an Antibacterial Platform for Soft Tissue

**DOI:** 10.3390/gels12060457

**Published:** 2026-05-23

**Authors:** Ioana Maria Marinescu, Andrada Serafim, Elena Olaret, Bogdan Stefan Vasile, Mona Mihailescu, Gratiela Gradisteanu Pircalabioru, Kristin Syverud, Stian Kreken Almeland, Samih Mohamed-Ahmed, Kamal Mustafa, Esko Kankuri, Cristian Botezatu, Bogdan-Stelian Mastalier-Manolescu, Alexandra Catalina Birca, Izabela-Cristina Stancu

**Affiliations:** 1 Advanced Polymer Materials Group, Faculty of Chemical Engineering and Biotechnologies, National University of Science and Technology POLITEHNICA Bucharest, 011061 Bucharest, Romania; ioana.marinescu2506@upb.ro; 2 Advanced Polymer Materials Group, CAMPUS Research Institute, National University of Science and Technology POLITEHNICA Bucharest, 060042 Bucharest, Romania; andrada.serafim0810@upb.ro (A.S.); elena.olaret@upb.ro (E.O.); 3 Research Center for Advanced Materials, Products and Processes, National University of Science and Technology POLITEHNICA Bucharest, 060042 Bucharest, Romania; bogdan.vasile@upb.ro; 4 National Research Center for Micro and Nanomaterials, Faculty of Chemical Engineering and Biotechnologies, National University of Science and Technology POLITEHNICA Bucharest, 060042 Bucharest, Romania; 5 Holographic Imaging and Processing Laboratory, Physics Department, National University for Science and Technology POLITEHNICA of Bucharest, 060042 Bucharest, Romania; mona.mihailescu@upb.ro; 6 Faculty of Biology, University of Bucharest, 050097 Bucharest, Romania; ggradisteanu@upb.ro; 7 RISE PFI, Department of Chemical Engineering, Norwegian University of Science and Technology (NTNU), NO-7034 Trondheim, Norway; kristin.syverud@rise-pfi.no; 8 Department of Plastic, Hand and Reconstructive Surgery, Norwegian National Burn Center, Haukeland University Hospital, NO-5021 Bergen, Norway; stian.almeland@uib.no; 9 Center of Translational Oral Research (TOR), Department of Clinical Dentistry, University of Bergen, NO-5009 Bergen, Norway; samih.ahmed@uib.no (S.M.-A.); kamal.mustafa@uib.no (K.M.); 10 Faculty of Medicine, University of Helsinki, 00100 Helsinki, Finland; esko.kankuri@helsinki.fi; 11 Surgery Department, University of Medicine and Pharmacy Carol Davila, 050474 Bucharest, Romania; cristian.botezatu@umfcd.ro (C.B.); bogdan.mastalier@umfcd.ro (B.-S.M.-M.); 12 Surgery Department, Colentina Clinical Hospital, 020125 Bucharest, Romania; 13 Department of Science and Engineering of Oxide Materials and Nanomaterials, National University of Science and Technology POLITEHNICA Bucharest, 011061 Bucharest, Romania; alexandra.birca@upb.ro; 14 Faculty of Medical Engineering, National University of Science and Technology POLITEHNICA Bucharest, 011061 Bucharest, Romania

**Keywords:** nanocellulose, hybrid material, reinforced hydrogel, mechanical properties, swelling kinetics, antibacterial activity

## Abstract

Modern wound care is challenged by the emergence of antibiotic-resistant bacterial strains, causing the need for advanced dressing materials that provide infection control while promoting healing. Although polyacrylamide (PAAm) hydrogels are widely investigated due to their biocompatibility, their lack of intrinsic antibacterial activity and poor mechanical properties restrict their clinical use. To overcome these limitations, this study proposes a natural–synthetic hydrogel that combines PAAm with TEMPO-oxidized cellulose nanofiber (TOCNF) functionalized silver nanoparticles (AgNPs). The synthesis is performed through the polymerization of the synthetic monomer in the presence of the TOCNF–AgNPs, the nanofibrillar cellulose simultaneously serving as a reducing and stabilizing agent for AgNPs, and as a plasticizer for the PAAm network. Morpho-structural analysis of the hybrid precursor (TOCNF–AgNPs) revealed two populations of AgNPs, offering a cumulative effect between rapid bacterial penetration and a prolonged ionic reservoir, while maintaining the stability of the system. The subsequent incorporation of the hybrid into PAAm matrix resulted in tunable swelling kinetics and mechanical properties. Wettability and surface stiffness improve with the increase in hybrid content. The antibacterial effect was confirmed by a colony-counting assay for formulations with higher AgNPs content, exhibiting inhibitory metabolic activity against several pathogenic strains. These results suggest that PAAm/TOCNF–AgNPs (PTA) nanocomposites represent a promising mechanically adaptive candidate for wound-care applications.

## 1. Introduction

Wound management, whether acute or chronic, remains a substantial challenge on a global scale. The therapeutic success is often limited by the patient’s compliance, treatment plans, and the materials used [1]. Conventionally, foams, hydrogels, and textile dressings are used as a barrier for microorganisms, hindering contact with the damaged area, irrespective of their involvement in the healing process [2]. Another aspect to consider when designing a wound dressing is the predisposition to infections. Nowadays, “superbugs” (i.e., methicillin-resistant *Staphylococcus aureus*) are more prone to infect tissue disruptions, making it harder to treat due to the increasing resistance to most antibiotics, leading to systemic manifestations [3,4]. Recent advancements led to the development of dressings that incorporate antibacterial agents [5,6], bioactive molecules [7,8], peptides [9,10], and nanocomplexes [11,12]. Wound management implies a multidisciplinary approach, combining the medical assessment of the wound and engineering the material according to the needs of the patient, synergizing the mechanical, chemical, and biological interactions between the material and the wound [13].

Because of their versatility, hydrogels have the potential to provide both structural support and preserve the favorable conditions needed to sustain the healing process. Their ability to absorb large quantities of biological fluids makes them particularly suitable for managing highly exudative wounds [14,15]. However, excessive swelling may also compromise dimensional stability and mechanical integrity, limiting long-term performance under physiological conditions [15,16].

In this context, PAAm emerges as an attractive candidate for wound dressing applications due to its high hydrophilicity, tunable mechanical properties, chemical stability, and biocompatibility [17,18]. The properties of PAAm-based hydrogels can be tuned by simply adjusting the ratio between components, making them suitable for applications ranging from soft-tissue fillers to contact lenses, up to wound dressings and drug delivery platforms [19]. Despite these advantages, the practical use of PAAm is still limited due to the lack of bioactivity and cell-adhesive functional groups, brittleness, and inadequate mechanical strength in the swollen state [20,21,22]. As a single-network hydrogel, PAAm is often too brittle, exhibiting insufficient mechanical resilience to withstand the dynamic physiological environment [14,23]. Consequently, recent studies [23,24,25] have explored the incorporation of reinforcing agents, particularly cellulose derivatives, to overcome these deficiencies.

Cellulose derivatives have attracted the attention of researchers as promising reinforcing agents due to their high aspect ratio, mechanical strength, and biocompatibility. Previous studies demonstrated that cellulose derivatives can significantly improve the mechanical performance of PAAm hydrogels by enhancing toughness, elasticity, and compressive resistance [24]. For instance, Yang et al. reported improved flexibility and toughness, while preserving the rubber-like elasticity of the PAAm network [25]. Similarly, Chuchu Chen et al. reported a 6.8-fold increase in compressive stress when compared to pristine PAAm and correlated to CNF load [23]. Although these studies demonstrated the reinforcing potential of cellulose, they mainly focused on mechanical optimization, while biological properties remained insufficiently investigated.

Among the cellulose derivatives, cellulose nanofibers (CNF) distinguish themselves through their flexible polymeric chains, which are organized in a fibrillar shape with a nanometric diameter ranging between 5 and 30 nm and a length reaching 1000 nm [26]. TEMPO-mediated oxidation further enhances their applicability by introducing carboxyl groups on the CNF surface. By generating TEMPO-oxidized cellulose nanofibers (TOCNF) with negative surface charges, the ion-binding ability is enhanced. These groups can also serve as nucleation sites for nanoparticle formation, making possible the fabrication of multifunctional hybrid systems for water purification, electrode coatings, and templates for catalytic composites [27,28]. Recently, Liu et al. developed a PAAm/TOCNF hydrogel containing AgNPs through a successive ion layer adsorption and reaction (SILAR) approach [29]. The TOCNF carboxylate groups served as coordination sites for Ag^+^, promoting nanoparticle nucleation and the PAAm network served as a carrier and protective microenvironment, hindering the oxidation of AgNPs. Overall, the material exhibited enhanced sensitivity for detecting hazardous compounds. However, the study did not report mechanical characterization, swelling kinetics, or the suitability of the hydrogel under physiological conditions, which are particularly important in biomedical applications.

Complementary studies emphasize the role of ion-mediated interactions acting as supramolecular crosslinking bridges in PAAm/TOCNF systems. By ionic crosslinking TOCNF with metallic ions (i.e., Ca^2+^), sacrificial bonds are created, effectively increasing the density of “load-bearing” junctions without changing the covalent PAAm connectivity. The supplementary reinforcement diminishes repulsive interactions, causing an increase in shear modulus and elastic friction, while toughening the microstructure [30,31]. In addition to the mechanical limitations, PAAm lacks intrinsic antibacterial properties; therefore, their effectiveness is significantly restricted in preventing bacterial colonization and biofilm formation. AgNPs hold the potential to enhance both the antibacterial activity and surface mechanical stiffness, as previous studies show [32,33]. AgNPs are distinguished by a superior antibacterial effect based on their dual mechanisms of action, involving both particle and ionic release. They anchor to the cell membrane, denaturing it, and are subsequently internalized, interacting with organelles from the cytoplasm, altering their structure, interfering with bacterial signal transduction pathways, and ultimately triggering cellular apoptosis. Simultaneously, AgNPs release silver ions (Ag^+^), which further bind to sulfur-containing proteins, DNA, and enzymes, ultimately inhibiting cellular metabolism and replication [34,35,36].

However, introducing metallic nanoparticles, such as AgNPs, in a hydrogel, remains challenging due to their tendency toward aggregation and increased chemical reactivity, leading to the opposite effect, and hindering the healing process [37].

Additionally, stabilizing the nanoparticles remains problematic, with common issues including the use of hazardous reagents, poor colloidal stability over time, and low synthesis yield [22,38]. Environmental concerns associated with the by-products of the process redirected the research towards more sustainable solutions. Various cellulose derivatives, including dialdehyde cellulose [39], bacterial cellulose [40], carboxymethyl cellulose [41], hydroxyethyl cellulose [42], hydroxypropyl methylcellulose [43], and TOCNF [44] have been effectively employed in the past decades to reduce silver precursors and stabilize the resulting nanoparticles simultaneously [44]. Compared with conventional approaches based on pre-formed AgNPs adsorbed onto cellulose substrates [29,45,46,47,48], in situ formation offers improved nanoparticle distribution, enhanced colloidal stability, and reduced dependence on hazardous reducing agents. These hybrid materials hold significant potential in various fields, serving, amongst others, as components of substrates for surface-enhanced Raman spectroscopy (SERS) [42], food-packaging layers [41], capping agents of heavy metal ions [44], antibacterial agents in wound dressings [49], and as a temporary cranioplasty mesh [50].

Although previous studies demonstrated the reinforcing potential of TOCNF [51] and the antibacterial effect of AgNPs, the simultaneous integration into a PAAm network remains insufficiently investigated in the context of wound management.

Therefore, the present work presents a two-step fabrication of a bioactive nanocomposite hydrogel comprised of PAAm, TOCNF, and AgNPs. Distinct from previous approaches, AgNPs were in situ synthesized on the TOCNF; subsequently, the hybrid material was immobilized in PAAm hydrogel via UV photopolymerization. Ionic bonds between carboxylic groups and calcium ions were created by immersing the hydrogel in calcium chloride solution (CaCl_2_), creating a dual crosslinked architecture. This method simultaneously facilitates the modulation of mechanical properties, ensures good control over AgNPs characteristics, and imparts antibacterial functionalities over the hydrogel matrix. To our knowledge, no such system has been investigated in the context of wound management.

## 2. Results and Discussion

### 2.1. Characterization of the TOCNF–AgNPs Colloid

**Figure 1:**
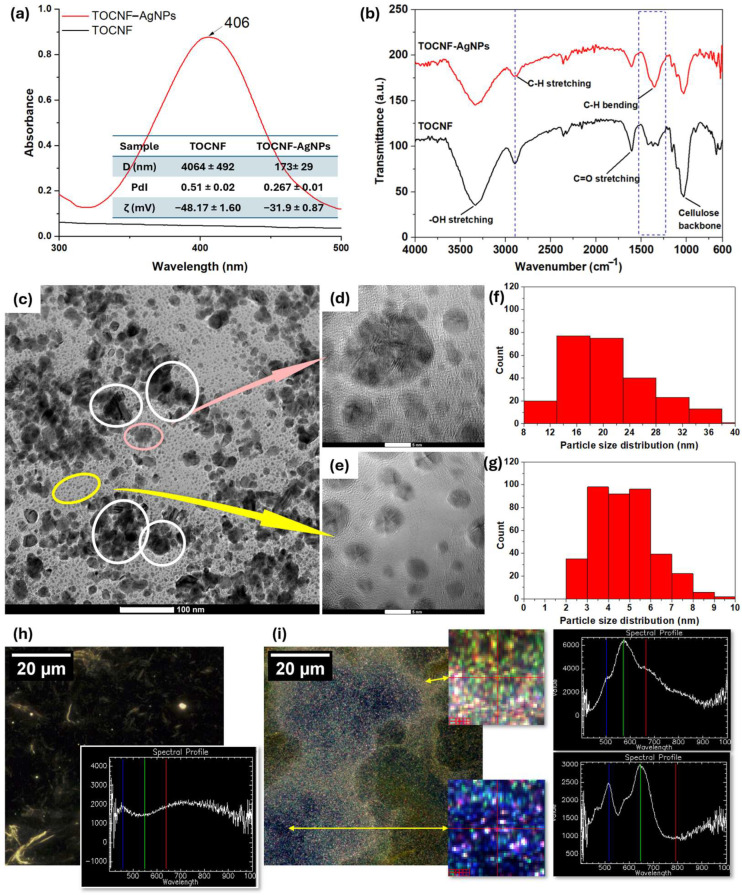
Confirmation of a successful synthesis of AgNPs: (**a**) UV–VIS spectra of pristine TOCNF compared with TOCNF–AgNPs (the insert table showing the DLS data); (**b**) ATR–FTIR spectra of pristine TOCNF and hybrid TOCNF–AgNPs; (**c**–**e**) TEM images show AgNPs distribution in pristine TOCNF; (**f**,**g**) histograms associated to the two distinct AgNPs populations; (**h**) EDHI of pristine TOCNF; and (**i**) TOCNF–AgNPs and spectral profiles of the central pixel from the regions indicated by the yellow arrows.

In addition to the color shift visible during the synthesis, the successful synthesis of the TOCNF–AgNPs hybrid was validated by UV–VIS spectroscopy. Previous research has shown that, in the case of spherical AgNPs, a surface plasmon resonance (SPR) band emerges between 380 and 460 nm [52,53]. As depicted in Figure 1a, a clear and sharp absorption band formed at 406 nm, attributed to small spherical nanoparticles. The insert represents the data collected by dynamic light scattering (DLS) analysis and exposes a decrease in dynamic particle diameter when silver nitrate (AgNO_3_) is added, possibly due to the formation of AgNPs and concurrent interaction with the TOCNF, reducing the electrostatic repulsion between the polysaccharide’s chains [48]. This statement is also supported by the polydispersity index (PdI) values, which, in the case of TOCNF–AgNPs, is 0.267, lower than the pristine TOCNF’s of 0.51. Hence, stability appears to be diminished with the presence of the AgNPs, whose nanometric dimensions are prone to aggregation. According to the literature, a TOCNF-based colloid is considered to be stable if its zeta potential (ζ) value is outside the range of ±30 mV [54]. After the functionalization, an increase in ζ, from −48 mV (pristine TOCNF) to −32 mV (TOCNF–AgNPs) was observed, indicating a reduction in electrostatic interactions; however, the system remains in the stability range.

Furthermore, the attenuated total reflectance–Fourier transform infrared (ATR–FTIR) spectra revealed evidence of interactions between the TOCNF nanofibers and the AgNPs. As shown in Figure 1b, the band assigned to the CH_2_ stretch shifts, when AgNPs are present in the system, from 2894 cm^−1^ to 2907 cm^−1^. A similar behavior was noticed for the stretching vibration band of C=O from the carboxylic salts at 1607 cm^−1^. The TOCNF spectrum exhibits characteristic peaks at 1423, 1370, and 1315 cm^−1^, associated with -CH_2_ scissoring, C-H bending and -CH_2_ wagging vibrations, respectively [55]. After in situ synthesis of AgNPs, they merge into a sharp peak at 1353 cm^−1^, probably as a result of the coordination between the nanoparticles and carboxylic groups [44]. These spectral changes support the dual function of TOCNF, which acts as both a stabilizing and reducing agent during nanoparticle formation.

Additional evidence for the successful formation of the TOCNF–AgNPs hybrids was provided by the transmission electron microscopy (TEM) micrographs (Figure 1c–e). The small nanoparticle diameters further support the quasi-spherical morphology of the AgNPs. Nanoparticle sizes were quantified using ImageJ 1.54g; the corresponding size distribution histograms are presented in Figure 1f,g. As can be observed, there are two groups of nanoparticles with quite distinct diameters: smaller, well-dispersed and larger, with many forming aggregates. Different areas were selected to measure the diameters. Most of the particle diameters ranged between 3 to 6 nm and 14 to 20 nm, respectively, displaying a monomodal distribution in each case. The AgNPs appear to be surrounded by thin amorphous regions originating from dehydrated TOCNF, indicating close association between the nanoparticles and the oxidized nanocellulose matrix. The dehydration during sample preparation prior to TEM analysis led to shrinkage of the hybrid when compared to the hydrated dimensions recorded by DLS. Furthermore, the aggregation of individual AgNPs observed in the micrographs (highlighted by white circles in Figure 1c), along with the length of the TOCNF, could also contribute to the larger average diameters measured by DLS [56,57]. Enhanced darkfield hyperspectral images (EDHI) and spectral profiles demonstrate the plasmonic properties of AgNPs existing inside TOCNF. In contrast to TOCNF, which has weak and diffuse fiber bundle scattering (Figure 1h), TOCNF–AgNPs have significant plasmonic heterogeneity (Figure 1i). While quasi-spherical particles are expected to contribute to the higher-energy (blue green) scattering component, closely spaced or fused particles within the aggregates supported coupled plasmon patterns, resulting in red-shifted maxima (from 535 nm to 575 nm) and spectral broadening. Spherical AgNPs generally exhibit a peak between 400 and 500 nm [58]. Thus, a strong correlation between the diameters of the nanoparticles and the spectrum profile is still not feasible in our instance, due to the high density and reduced dimensions of AgNPs [59].

### 2.2. Crosslinking Efficiency in PAAm-Hydrogels

**Figure 2:**
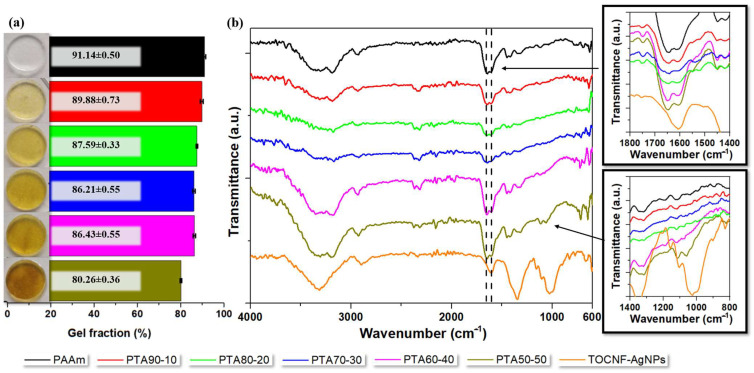
Gel fraction values and digital images of the samples post-extraction (**a**); and ATR–FTIR spectra (**b**) for both control (PAAm) and composite hydrogels with interest regions zoomed.

The gel fraction analysis confirmed that the synthesis protocol produced efficiently crosslinked materials. Nevertheless, composite hydrogels exhibited lower gel fraction values compared to the pristine PAAm system. As illustrated in Figure 2, a progressive decrease in gel fraction was observed with decreasing PAAm content and increasing TOCNF–AgNPs loading, from PTA90-10 to PTA50-50, respectively. This trend likely reflects the reduced ability of the synthetic network to effectively stabilize the TOCNF-based hybrid, whose structural dimensions and rigidity may hinder complete incorporation into the polymer matrix. Additionally, while abundant carboxylic groups lead to improved water retention, excessive swelling can overexpand the polymer network and, supposedly, diminish the stability and the mechanical properties of the hydrogel [60], thereby contributing to the decrease in the gel fraction.

The characteristic PAAm bands are present in the range of 1700–1500 cm^−1^, associated with the C=O stretching vibration and N-H bending vibration [29]. In the same region, TOCNF exhibits a peak at 1600 cm^−1^, corresponding to the stretching vibration of the C=O bond from the carboxylic salts [61]. The shift of the C=O vibration from 1646 to 1648 cm^−1^ and N-H bending from 1607 to 1602 cm^−1^ can be assigned to a potential H-bonding interaction at the interface PAAm with the nanofibrillar TOCNF phase, as reported in [14,62]. The 1200–900 cm^−1^ region, highlighted in Figure 2b, shows an alteration in the spectral contour of the C–O–C and C–O vibrations characteristic of TOCNF. The broadening of the specific band confirms the incorporation of cellulose into the hydrogel network [63]. The band attributed to CH_2_ stretching vibration appears at 1418 cm^−1^ [14].

### 2.3. Affinity for Aqueous Media

**Figure 3:**
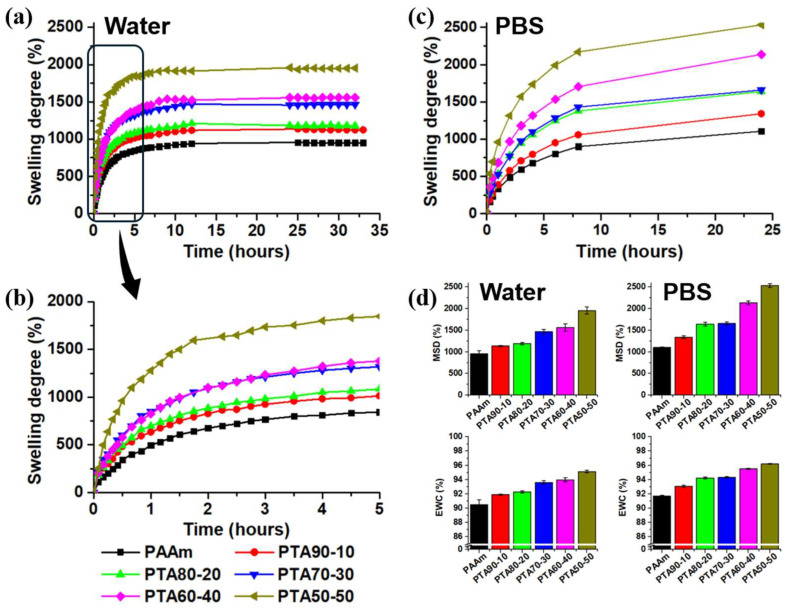
Water affinity of the hydrogels: (**a**,**b**) swelling kinetics of samples in water; (**c**) swelling behavior of hydrogels in PBS; and (**d**) maximum swelling degree and equilibrium water content for both water and PBS testing.

The hydrogels’ affinity for aqueous media was assessed and correlated with their composition (Figure 3). All samples reached hydration equilibrium within 27 h and displayed a rapid swelling phase during the first 4 h (Figure 3a,b). Both the swelling rate and the maximum swelling degree (MSD) increased with increasing cellulosic content: while the pristine PAAm hydrogel exhibited an MSD value of only 958.02 ± 72%, the highest value (1957.98 ± 80%) was recorded for sample PTA50-50 (Figure 3c). The enhanced affinity for aqueous media can be primarily attributed to TOCNF, due to the presence of hydrophilic groups (hydroxyl and carboxyl groups), while fibers modify the hydrogel structure, facilitating matrix expansion. A similar trend was observed for the equilibrium water content (EWC), with all hydrogels exceeding 90% and showing a slight increase as the TOCNF–AgNPs concentration increased (Figure 3c).

Analysis of the swelling kinetics revealed non-Fickian diffusion for all samples, with n values between 0.5 and 1, additionally indicating that network relaxation due to TOCNF–AgNPs loading plays an essential role in the swelling process (Table 1). While the diffusion rate is not directly affected by the hybrid content, clear differences were observed in both swelling magnitude and kinetics. The presence of the TOCNF–AgNPs hybrid generates a more flexible network structure, enabling higher water uptake.

Formulations were also tested in phosphate-buffered saline (PBS); the results are presented in Figure 3c,d. When immersed in PBS, PTA hydrogels exhibited a higher swelling rate, achieving equilibrium, within 24 h, of 2533 ± 40% (PTA50-50).

The higher swelling in PBS is assigned on one hand to the deprotonation of carboxylic groups from TOCNF, resulting in increasing negative charge and leading to electrostatic repulsions [64,65]. When the pH of the medium is higher than the pKa of the carboxylic groups (2.8–3.7 [64]), the deprotonation of the -COOH is enhanced, increasing the negative charge of the TOCNF. This effect can be seen in our results as well (Figure 3c,d). At a 7.4 pH value, the MSD% increases 1.29-fold when compared to the water pH of 6.4. Despite the interactions that occur between these groups and the counterions from the PBS, the enhancement of the swelling remains clearly observable. Moreover, the increase in MSD for the PAAm control hydrogel can be assigned to the hydrolysis of some of the amide groups into carboxylic acid. This process additionally contributes to the increase in swelling from the PTA hybrids [66].

### 2.4. Assessment of the Mechanical Behavior of the Hydrogels at Macroscale

Amplitude sweep tests were performed to determine the linear viscoelastic region (LVR) and the yield point of the hydrogels. All samples exhibited a well-defined LVR at low strain values, where storage modulus (G′) remained constant and dominant over loss modulus (G″), indicating elastic behavior. Beyond a critical strain value, both moduli started to decrease, reflecting the progressive disruption of the hydrogel network structure. The yield point, defined as the intersection between G′ and G″, occurred between approximately 10% and 30% of the strain, depending on the PAAm content. Samples with a higher content of polymer (i.e., PAAm and PTA90-10) showed higher G′ values and a slightly extended linear region, indicating stronger and more stable network structures. In contrast, samples with lower PAAm content displayed reduced modulus values and an earlier transition to viscous behavior, suggesting a weaker crosslinked network. Overall, the results highlight a clear dependence of the viscoelastic response on polymer concentration. The variation in G′ and G″ as a function of strain is presented in Figure S1.

**Figure 4:**
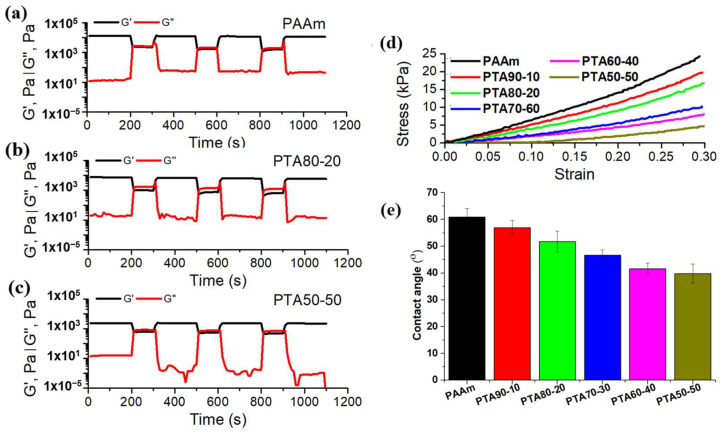
The self-recovery behavior of the materials as plotted following rheological testing: (**a**) PAAm; (**b**) PTA80-20; (**c**) PTA50-50; (**d**) the mechanical characteristics of the hydrogels, determined through uniaxial compression; and (**e**) dependency surface wettability composition, as reflected from the average contact angle values.

Frequency sweep measurements were carried out within LVR to investigate the viscoelastic behavior of the hydrogels as a function of frequency (Figure S2). In all cases, G′ was significantly higher than G″ throughout the tested frequency range, confirming the predominance of the elastic component and the formation of well crosslinked, stable networks, in agreement with the results registered in the gel fraction study. At low frequencies, both moduli remained nearly constant, indicating that the hydrogel structure resists deformation and maintains its integrity under slow oscillatory stress. At higher frequencies, a slight increase in both G′ and G″ was observed, suggesting the restricted mobility of the polymer chains and a more pronounced elastic response. Samples with higher synthetic polymer content (i.e., PAAm and PTA90-10) exhibited larger modulus values, reflecting denser and more cohesive networks, whereas samples with lower PAAm concentrations (i.e., PTA60-40 and PTA50-50) showed smaller moduli and a softer mechanical profile, probably due to the prevalence of physical interactions between cellulose fibrils rather than strong covalent crosslinking, resulting in softer and less stable structures. These results demonstrate that the mechanical stiffness and structural stability of the hydrogels are strongly dependent on polymer concentration, in good agreement with the amplitude sweep findings. Furthermore, the self-recovery tests revealed that all compositions exhibit reversible viscoelastic behavior, demonstrating a capacity for structural rebuilding once the high strain is removed (Figure S3). Samples PAAm and PTA90-10 displayed the typical response of crosslinked PAAm networks, characterized by a stable G′ (Figure 4a). Because their structure is dominated by covalent bonding, the increase in G″ observed after the first high-strain step can be attributed to the irreversible rupture of some crosslinks. In these samples, the covalent backbone remains largely intact, yet the overall structural recovery is incomplete. In contrast, samples PTA80-20 and PTA70-30 showed a more efficient recovery for both G′ and G″, suggesting a more balanced interplay between covalent and physical interactions that allows for the reformation of the network (Figure 4b). For the PTA60-40 and PTA50-50 samples, the initial rise in G″ during the high-strain step indicates an enhanced energy dissipation capacity, likely resulting from increased macromolecular mobility (Figure 4c). However, this ability diminishes after subsequent cycles, possibly due to chain entanglement and progressive structural fatigue.

Uniaxial compression testing provides valuable insight into the reinforcement mechanisms generated by incorporating the hybrid material into the PAAm matrix. The stress–strain curves (Figure 4d) exhibit the characteristic nonlinear profile of polymeric networks: an initial linear region associated with elastic deformation, followed by strain hardening at higher deformations (~0.08–0.12) as the polymer chains begin to align under load. Young’s modulus decreases with the increase in the content of TOCNF–AgNPs, as shown in Table 1. Although TOCNF can act as a reinforcing phase, excessive incorporation may disrupt the continuity of the PAAm network, leading to a softer and more compliant structure [62]. The results are consistent with the rheology testing data, showing a gradual decrease in modulus with increasing TOCNF–AgNPs content, indicating a shift towards a more deformable TOCNF-dominated network. Notably, despite the lower stiffness, the TOCNF-containing hydrogels exhibited improved deformation tolerance compared to neat PAAm, suggesting that the hybrid network favors flexibility and energy dissipation over rigidity.

### 2.5. Mechanical Behavior of the Hydrogels at Microscale

The results obtained confirmed the elastic-dominant behavior for all hydrogels with G′ > G″ (Table 2). An increase in G′ with increasing the PAAm content is observed from sample PTA50-50 to PTA80-20, with the lowest value for sample PTA50-50 and the highest value for sample PTA80-20. A slight decrease in G′ is observed for sample PTA90-10 when compared to both sample PTA80-20 and the control PAAm sample. Higher values of G″ for sample PTA90-10 and PAAm suggest that these samples have a more pronounced viscoelastic contribution due to a more relaxed hydrogel network with the lowest reinforcing effect. On the other hand, the lowest value of G″ was obtained on sample PTA70-30, suggesting a reduced viscous behavior, probably due to a network with restricted chain mobility.

Overall, the mechanical features are strongly influenced by the PAAm:TOCNF–AgNPs ratio at both the macroscale and microscale. As previous research has demonstrated, the addition of TOCNF significantly contributes to the mechanical integrity of the hydrogel through physical entanglement and hydrogen bonding with PAAm chains [67]. The carboxylate groups on TOCNF further enhance interfacial adhesion and facilitate ionic interactions, leading to improved stiffness and energy dissipation under compression [67]. These interactions generate an interpenetrating network that better resists deformation, especially when multivalent cations are involved [68]. At the microscale, maximum elasticity was recorded for PTA80-20, while maintaining a moderate viscous dissipation, indicating optimal network reinforcement. This system also exhibited the best self-recovery ability, as demonstrated by the rheological testing.

### 2.6. Contact Angle

PAAm is intrinsically hydrophilic, with reported contact angle values between 30° and 80° [69,70,71]; however, its measurable hydrophilicity, expressed through parameters such as water uptake or surface wettability, depends on structural features of the network and hydration state, including crosslinking density and the incorporation of additional hydrophilic constituents such as TOCNF. Average contact angle values diminish with the increase in hybrid content, as shown in Figure 4e, leading to a more hydrophilic hydrogel surface. Owing to its high affinity for water and the abundance of carboxyl groups, TOCNF enhances both the surface wettability and the bulk hydration capacity of the material [72]. This is consistent with our findings, obtained following swelling and mechanical tests, where the hybrid not only increases network flexibility and water uptake but also seems to promote more efficient hydrogen bonding due to the presence of -COOH functionalities [73]. Moreover, the improved surface wettability can be attributed to increased surface free energy generated by the TOCNF-rich regions. The polar carboxyl groups facilitate the rapid establishment of hydrogen bonds with water molecules, supporting the formation of a stable hydration layer at the interface [74].

### 2.7. TGA

**Figure 5:**
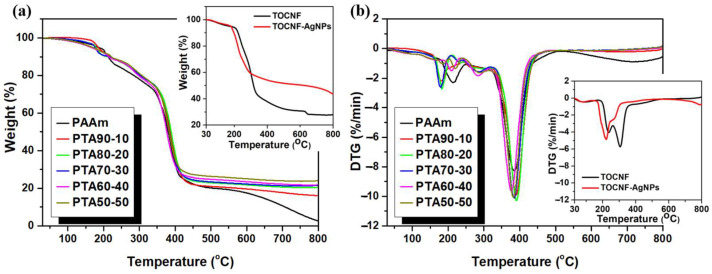
(**a**) TGA; and (**b**) DTG registered for PTA hydrogels and TOCNF–AgNPs (inserts).

The thermal stability of the nanocomposite was determined in the temperature range 30–800 °C to explore the influence of the hybrid. The thermal decomposition occurred in stages: three for PAAm and four for the composite systems (Figure 5a). The initial stage occurred between 0 and 170 °C, correlating with the removal of water, indicated by a minor mass reduction on the thermogram [75]. The second stage, at 170–280 °C, is related to the gradual degradation of the amide groups of the PAAm [76]. When compared to pristine PAAm, this peak becomes sharper and more pronounced in composite hydrogels (Figure 5b). It is displaced to lower temperatures, as shown in Table S1, most likely due to deterioration of the amorphous sections in TOCNF structure, which occurs in the same temperature range. A smaller peak develops in the third region, indicating the breakdown of TOCNF’s glycosidic bonds from the crystalline phase. The final stage (380–420 °C) is characterized by the complete destruction of the PAAm’s polymeric chains, as well as the formation of an anhydride by the elimination of water molecules from the carboxylic groups, as indicated by the increased residual mass (Table S1) [76,77]. Although AgNPs improve the hybrid’s thermal stability, they provide no evident benefit in the case of composite hydrogels other than contributing to the carbonized matrix of the cellulose, which results in a high residual mass percentage.

### 2.8. Evaluation of Bacterial Viability and Metabolic Activity

Metabolic activity of bacterial cells was evaluated by XTT assay and expressed as optical density (OD) values, reflecting the residual metabolic activity after exposure to the tested hydrogels. Bacterial viability was quantified by colony-forming unit (CFU) counting and expressed as CFU/mL.

Compared to the untreated control, which is considered to have 100% viability, two of the tested hydrogels (PTA50-50 and PTA60-40) caused a significant reduction in the number of *Staphylococcus aureus (S. aureus)* cells, decreasing viability to approximately 40–60%. They appear to possess an effective antibacterial profile due to the adequate release of silver ions or optimal exposure of the nanoparticles. Samples with a lower content of TOCNF–AgNPs presented a moderate inhibitory effect against bacteria with a decrease in viability of 70–90%, indicating present, but reduced, antibacterial activity. For PTA90-10 and PAAm hydrogels, the viable cells were similar to the control sample, suggesting an insufficient concentration of AgNPs.

**Figure 6:**
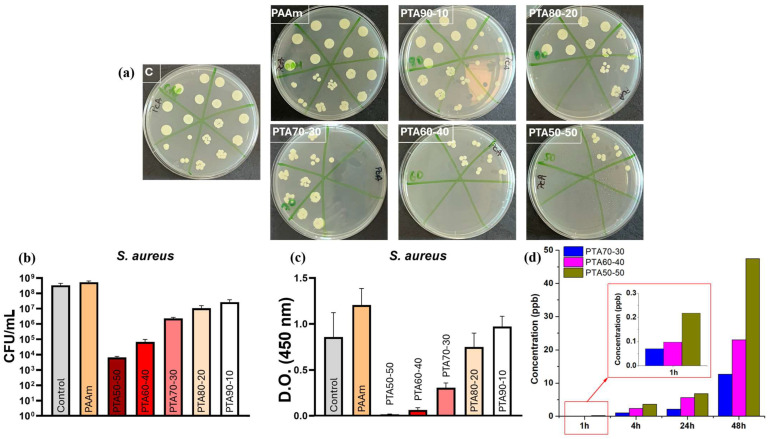
(**a**) Digital images of *S. aureus* cultures after 24 h of incubation in presence of hydrogels; (**b**) number of viable cells expressed as CFU/mL; (**c**) XTT assays results; and (**d**) ICP–MS results of the most efficient samples after 1, 4, 24 and 48 h after immersion in filtered water at 37 °C.

The XTT assay showed strong differences among the analyzed hydrogels, reflecting the residual metabolic activity of *S. aureus* following exposure to the tested materials (Figure 6c). Compared to the control, which presented a moderate level of XTT reduction, pristine hydrogel generated the highest absorbance, suggesting, as expected, increased metabolic viability and even a possible stimulation of bacterial proliferation.

Samples PTA50-50 and PTA60-40 presented extremely low values of the XTT signal, indicating a near-complete inhibition of the metabolic activity. This observation is in accordance with the CFU data previously presented, suggesting a strong antibacterial effect. Sample PTA70-30 displayed reduced, but detectable, metabolic activity indicating a moderate inhibitory effect, while hydrogels PTA80-20 and PTA90-10 showed significantly higher XTT intensity, corresponding to an increased metabolic activity. The PTA90-10 hydrogel aligns to the control profile, suggesting minimal inhibition. The antibacterial performances of these hydrogels are mainly influenced by the concentration and proper distribution of AgNPs within the network and Ag^+^ release, respectively. Poor distribution or low concentration of nanoparticles leads, as other research shows, to partial inhibition of the strain [78,79]. The ICP–MS analysis (Figure 6d) demonstrated a concentration-dependent release profile of Ag^+^, which strongly correlated with the TOCNF–AgNPs content. Enhanced release efficiency resulted in a higher accumulation of Ag^+^ in the medium and, consequently, the most potent antibacterial effect. It is also known that TOCNF presents an antifouling effect against bacteria membrane, which leads to a hindered adhesion of the biofilm to the materials [80]. Presumably, this property also contributes to the overall antimicrobial efficacy of these hydrogels. By combining the biocidal effect of AgNPs with the repulsive forces exhibited by TOCNF toward bacterial membranes, the otherwise proliferation-supporting pristine PAAm hydrogels become an effective material with antibacterial potency.

**Figure 7:**
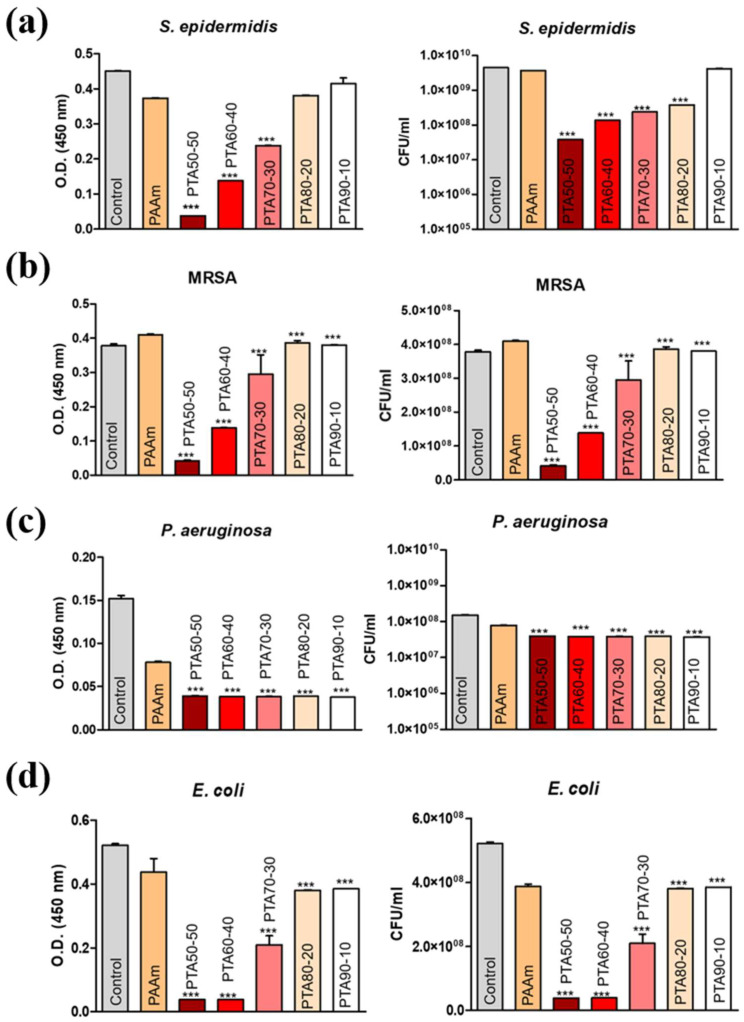
Antibacterial activity of the developed hydrogels against different bacterial strains after 24 h of incubation: (**a**) *S. epidermidis*; (**b**) MRSA; (**c**) *P. aeruginosa*; and (**d**) *E. coli*. *** indicates statistical significance at *p* < 0.001.

Additionally, the antimicrobial activity of the developed hydrogels was evaluated against other clinically relevant strains, including methicillin-resistant *Staphylococcus aureus* (MRSA), *Staphylococcus epidermidis* (*S. epidermidis*), *Pseudomonas aeruginosa* (*P. aeruginosa*), and *Escherichia coli* (*E. coli*), as presented in Figure 7. The results indicate that the antibacterial efficacy is strain-dependent, with a more pronounced inhibitory effect observed for Gram-positive bacteria, particularly MRSA, and notable activity against *P. aeruginosa*, a pathogen well known for its resistance and biofilm-forming capacity.

Overall, the concordance between CFU enumeration and XTT assay results confirms that hydrogels PTA50-50 and PTA60-40 exhibit the strongest and most consistent antibacterial activity across multiple strains. The observed differences between formulations likely arise from variations in composition, which influence the release of active components and the ability to induce bacterial membrane damage and metabolic disruption. Ag^+^ interacts electrostatically with the negatively charged membrane while small AgNPs penetrate through pores and channels [81]. Gram-positive bacteria present a thick peptidoglycan membrane easily disrupted and with greater intracellular exposure, while Gram-negative bacteria have an extra lipopolysaccharide membrane restricting the contact with AgNPs. Due to the more complex structure, the effect of AgNPs against Gram-negative bacteria is limited by the nanoparticle and ion release capacity of the material [82,83]. A close contact with the infected region and a proper concentration of the AgNPs proved to be efficient against *E. coli*. However, the dual membrane of *P. aeruginosa* proved to limit the penetration and overall effectiveness of the nanocomposite.

### 2.9. Evaluation of Biocompatibility

**Figure 8:**
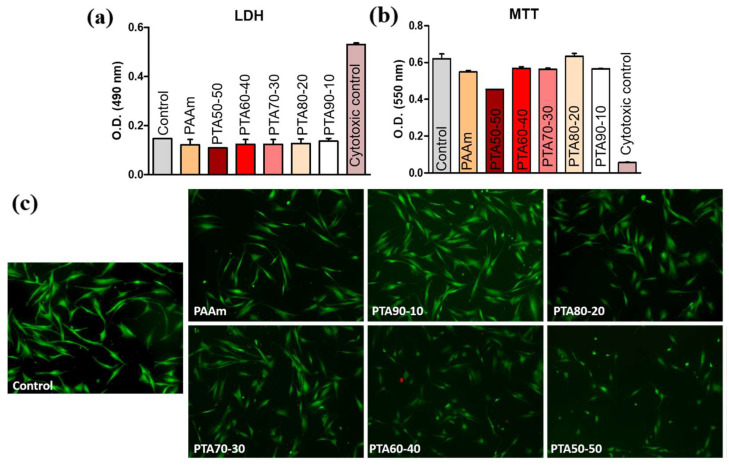
Biocompatibility evaluation on human dermal fibroblasts (HDF cell line): (**a**) cell viability assessed by MTT assay, expressed as OD at 550 nm, reflecting mitochondrial metabolic activity; (**b**) cytotoxicity evaluated by LDH release assay, expressed as OD at 490 nm, indicating loss of membrane integrity; and (**c**) representative fluorescence microscopy images from live/dead staining, where viable cells are stained green (calcein-AM) and dead cells are stained red (ethidium bromide), illustrating cell morphology and viability.

The cytocompatibility of the developed hydrogels was evaluated on human dermal fibroblasts (HDF). The MTT assay results (Figure 8a) indicate that the hydrogels support fibroblast metabolic activity, with most samples exhibiting viability levels comparable to, or higher than, the control. This suggests that the materials do not impair mitochondrial function and may even promote cellular activity, likely due to their favorable physico–chemical properties and hydrophilic nature.

The LDH assay (Figure 8b) demonstrated low levels of enzyme release for all tested hydrogels, indicating minimal membrane damage and limited cytotoxic effects. Although slight variations between samples were observed, these differences remain within acceptable limits for biocompatible materials, confirming that the hydrogels do not induce significant cell lysis.

Live/dead staining (Figure 8c) further supports these findings, revealing a predominance of viable (green) fibroblasts with normal elongated morphology and good spreading on the hydrogel surfaces. The low number of dead (red) cells indicates that the materials provide a supportive microenvironment for cell adhesion and proliferation.

Overall, the combined results from MTT, LDH, and live/dead assays demonstrate that the developed hydrogels exhibit good cytocompatibility toward HDF cells, maintaining high viability and low cytotoxicity. These properties, together with their previously demonstrated antibacterial activity, highlight the potential of these hydrogels for applications in wound healing and skin tissue engineering, where both antimicrobial performance and host cell compatibility are essential.

## 3. Conclusions

We report the development of bioactive PAAm-based nanocomposite hydrogel using a two-step strategy in which TOCNF modulates the mechanical properties, improving the resilience and flexibility of the matrix, while AgNPs provide antibacterial characteristics. First, the TOCNF–AgNPs hybrid was synthesized, resulting in a stable, well-dispersed system. ATR–FTIR confirmed the in situ generation of AgNPs on the TOCNF; TEM revealed two nanoparticle populations with distinct mean diameters. These populations most likely contribute to the overall antibacterial properties through complementary mechanisms: (1) the direct effect of the nanoparticles on the cells; and (2) an ion reservoir for Ag^+^ release.

Second, the hybrid was integrated in PAAm network, showing only subtle changes at the molecular level on ATR–FTIR spectra. Although UV-induced crosslinking was partially hindered by the TOCNF–AgNPs hybrid, the swelling degree increased with TOCNF–AgNPs content, indicating enhanced network relaxation in aqueous media at 37 °C. Mechanical testing highlighted a shift toward a more compliant network, with Young’s modulus decreasing as TOCNF–AgNPs increased. Frequency sweep measurements suggested that TOCNF promotes macromolecular mobility, enabling partial network reformation after stress is applied. Hence, structural fatigue seems to appear in higher TOCNF content samples.

Overall, the PAAm:TOCNF–AgNPs ratio modulates the compressive stiffness and energy dissipation at the surface through chain entanglement, hydrogel bonding and ionic interactions, with the optimal reinforcement observed in the PTA80-20 sample. Despite the presence of AgNPs, the higher TOCNF–AgNPs content enhances the hydrophilicity of the hydrogel, as shown by lower average contact angle values. As expected, higher TOCNF–AgNPs content leads to more efficient antibacterial effect when tested against *S. aureus*, PTA50-50, and PTA60-40, nearly fully inhibiting bacterial metabolic activity.

Collectively, the data presented in this study suggest that PTA hydrogels with an increased content of TOCNF–AgNPs may be an attractive alternative for wound management due to their flexibility, sustained antibacterial functionality, and improved water retention. Hence, additional studies, such as long-term release of Ag^+^ and swelling behavior, might be considered for further research.

## 4. Materials and Methods

### 4.1. Materials

A 1% solid content TOCNF dispersion was prepared at RISE PFI, Norway, as previously described [84]. Silver nitrate (AgNO_3_) was procured from VWR Chemicals (Radnor, PA, USA). Sodium hydroxide (NaOH) pallets, acrylamide (AAm), N,N′-methylene-bis-acrylamide (MBA), and Irgacure 2959 powders were acquired from Sigma Aldrich (St. Louis, MO, USA). The photoinitiator was used at 10% (w/v%) in ethanol. For metabolic activity determination, the CyQUANT XTT Cell Viability Assay Protocol kit (Thermo Fisher Scientific, Waltham, MA, USA) was used. Nutrient broth (TSB) was procured from Thermo Scientific. The MTT Cell Proliferation Assay Kit and LDH Cytotoxicity Detection Kit were procured from Roche Diagnostics. All reagents were used as received, without any further purification.

### 4.2. Synthesis of the TOCNF–AgNPs Hybrid

The practical steps of the TOCNF–AgNPs hybrid synthesis were performed following a similar protocol as that described in [44]. Briefly, TOCNF dispersion was diluted in distilled water to a 0.2% concentration at 40 °C under continuous stirring for 1 h. AgNO_3_ was added to the dispersion in a 1:2 mass ratio with respect to the dry weight of TOCNF and stirred for 3 h. A freshly prepared solution of 1 M NaOH was dropwise added to the mixture to accelerate the generation of AgNPs (AgNO_3_:NaOH = 0.6 molar weight). After 1 h, the obtained hybrid was cooled at room temperature and stored at 4 °C until further use.

### 4.3. Characterization of TOCNF–AgNPs Hybrid

To explore the successful formation of the nanoparticles, several analyses were conducted. UV–VIS spectroscopy was performed using a Shimadzu UV-3600 spectrophotometer (Kyoto, Japan) to verify and assess the plasmonic behavior of AgNPs in the presence of TOCNF within the 200–600 nm wavelength range. Samples were previously diluted with distilled water to minimize the light-scattering effect. Distilled water was used as a blank.

ATR–FTIR spectrometry was performed using a Jasco 4200 spectrometer (JASCO Deutschland GmbH, Pfungstadt, Germany) equipped with a Specac Golden Gate ATR device (Specac Ltd., Orpington, UK) at a 4 cm^−1^ resolution. The analyses were performed using a sapphire anvil on dried samples, in the wavenumber interval 4000–600 cm^−1^, with 200 scans/sample.

The hydrodynamic diameter (D), PdI, and ζ of the TOCNF–AgNPs nanoparticles were measured by DLS (Zetasizer Nano ZS, Malvern Panalytical, Worcestershire, UK), equipped with an He–Ne linear polarized laser. The scattering angles at which measurements were made were 173° and 13° for ζ and D, respectively.

For further examination, TEM was performed using a HR-S/TEM Titan Themis 200 (Thermo Fisher Scientific, Hillsboro, OR, USA) with a Schottky Field (X-FEG) emission source at 200 kV. The measurements were made using Digital Micrograph (Gatan Ametek, Pleasanton, CA, USA) software. The images acquired were obtained at a high resolution of 4 k × 4 k pixels. Prior to analysis, the sample was freeze-dried. For particle size diameter, ImageJ 1.54g software was used. At least 250 measurements were performed.

### 4.4. Hydrogel Synthesis

A 10% AAm solution (w/v) was prepared in distilled water, and MBA was added (2:100 molar ratio with respect to AAm) as crosslinker. After dissolution, the corresponding volumes of hybrid were added to the pre-polymerization solution, at 40 °C, resulting in the formulation described in Table 3. The TOCNF–AgNPs volumes were calculated with respect to the AAm and MBA solution.

A 10% Irgacure 2959 solution in ethanol was used as photoinitiator in a mass ratio of 1:100 with respect to AAm. The compositions were cast between 2 glass plates with a gap of 1 mm and in Petri dishes, respectively, and polymerized on an ECX-F26.M transilluminator (Vilber Lourmat, Marne la Vallée France) at 312 nm wavelength and 70% intensity. Samples were placed at a distance of 4 cm to the device and were subjected to 2 cycles of crosslinking of 15 min each.

Additionally, the samples were soaked in 2% CaCl_2_ solution (w/v) for TOCNF crosslinking for 30 min on an orbital shaker. Samples were washed thrice in distilled water to eliminate unreacted species.

### 4.5. Characterization of Nanocomposite Hydrogels

#### 4.5.1. Polymerization Efficiency

The efficiency of UV crosslinking was estimated through a gel fraction test using Equation (1), as described in [85]. After synthesis, samples were placed in an oven at a constant temperature of 37 °C until complete dehydration (*w_i_*). They were weighed, purified through repeated washings with distilled water, dried in the oven at 37 °C, and then weighed again (*w_f_*). The test was performed in triplicate.

(1) $$Gel\;fraction\;(\%)=\frac{w_{f}}{w_{i}}\times 100$$

#### 4.5.2. ATR–FTIR

The composite hydrogels were analyzed by ATR–FTIR spectroscopy using the same device and technique from 4.3. The obtained ATR–FTIR spectra were compared to the spectral appearance of the control PAAm hydrogel to identify the characteristic vibrational bands of both PAAm and cellulose, thereby confirming the incorporation of the hybrid components into the polymer network.

#### 4.5.3. Swelling Kinetics

The water affinity was assessed through gravimetric swelling assessment to investigate the influence of the hybrid upon PAAm hydrogels. Samples were cut with a stainless-steel hole puncher into discs with a diameter of 8 mm and a height of 3 mm, dried, and weighed before testing. Each sample was immersed in 30 mL of distilled water at 37 °C. The conventional gravimetric method was applied to register the mass changes at predefined time intervals. SD and EWC were calculated using Equations (2) and (3). MSD (% is considered the swelling degree of the compositions when hydration equilibrium was reached):

(2) $$SD\;(\%)=\frac{w_{f}-w_{i}}{w_{i}}\times 100,$$

(3) $$EWC\;(\%)=\frac{w_{f}-w_{i}}{w_{f}}\times 100$$

where *w_f_*—weight of hydrated sample, *w_i_*—initial weight of the dry sample.

Water diffusion was also evaluated based on Equation (4). The diffusion exponent (*n*) was obtained from the slope of the linear region from the logarithmic swelling fraction and time until the swelling reached an absorption of the solvent of 60% into the hydrogel structure, as presented in [86]:

(4) $$f=\frac{S_{t}}{S_{e}}=Kt^{n}$$

where *f*—swelling fraction, *S_t_*—water content at time *t*, *S_e_*—water content at equilibrium, *K*—hydrogel network dependent constant.

Hydrogel swelling behavior was also evaluated in PBS (pH 7.4) at 37 °C, maintaining a ratio of 4 mL of buffer per 10 mg of dry sample. The MSD and EWC were calculated according to the previously mentioned methods [85].

#### 4.5.4. Assessment of the Mechanical Behavior of the Hydrogels at Macroscale

To evaluate the mechanical characteristics of the materials at macroscale, both rheological and classical uniaxial compression tests were performed. First, measurements were carried out using a Kinexus Pro rheometer (Malvern, Worcestershire, UK) equipped with a serrated parallel-plate geometry (20 mm upper plate diameter) and a Peltier element for precise temperature control. Samples were placed on the lower plate and the upper plate was lowered until a normal force of 0.5 N was reached. All tests were conducted at 34 °C to mimic skin temperature. The LVR was first determined by performing an amplitude sweep at a frequency of 1 Hz within a stress range of 1–1000 Pa. Subsequently, a frequency sweep was performed to evaluate the material response across the 0.1–100 Hz range, under a constant stress of 1 Pa (within the LVR). Finally, the self-recovery behavior was investigated through a strain step test, alternating between a low strain (within the LVR) and a high strain (above the yield point) over three consecutive cycles. Throughout all measurements, a stainless-steel water-lock hood was used to prevent sample dehydration.

Unconfined uniaxial compression tests were performed on samples with similar diameters (3.21 ± 0.39 mm) and heights (8.26 ± 0.19 mm using a Brookfield CT3 texture analyzer (Middleboro, MA, USA). Samples were analyzed in the equilibrium–swollen state by being placed on the lower plate of the equipment and compressed by TA25/1000 accessory. The speed of compression was 0.05 mm/s up to a 30% deformation at a trigger load of 0.005 N. Young’s modulus was determined in the linear region of the stress–strain curve.

#### 4.5.5. Evaluation of the Mechanical Features at Microscale

Local viscoelastic properties were assessed through instrumented indentation using a G200 Nanoindenter (KLA Instruments, Milpitas, CA, USA) and a flat-punch diamond tip with a diameter of 511.36 nm. G′ and G″ were obtained using the “G-Series DCM CSM Flat Punch Complex Modulus, Gel” method implemented in the NanoSuite 6.52.0 software. To ensure full contact between the tip and the sample, the pre-compression test was set to 20 µm. An oscillation amplitude of 100 nm was applied with a frequency of 10 Hz. Seven indentations were performed on equilibrium hydrated samples, ensuring at least 2 mm between them. Results were reported as mean ± standard deviation, setting Poisson’s ratio to 0.5.

#### 4.5.6. Surface Wettability of the Hydrogels

Hydrogels’ wettability was assessed using the sessile drop technique with Drop Shape Analyzer 100 (DSA 100, KRÜSS GmbH, Hamburg, Germany). Membrane cast samples were dried directly on the microscope slide at room temperature. At least 3 different plane regions of each sample were selected for testing. Drops of 2 μL distilled water were placed on the dried samples, contact angle measurements being recorded for 60 s. The data presented in Figure 4e represents the overall mean contact angles ± standard deviations, calculated from the computed average given by the instrument’s software (ADVANCE 1.7.2.1) [87].

#### 4.5.7. Thermal Analysis

Thermogravimetric analysis was performed using NETZSCH TG 209 F1 Libra equipment (Selb, Germany). Samples of approximately 10 mg (±2 mg) were heated gradually from 30 °C to 800 °C with a 10 °C heating rate under a nitrogen atmosphere with a flow rate of 20 mL/min.

#### 4.5.8. Inductively Coupled Plasma–Mass Spectrometry (ICP–MS) Analysis

Dried samples were immersed in distilled water and maintained at 37 °C. The distilled water was pre-filtered using a 0.22 µm membrane filter to ensure purity. 2 mL of each medium was extracted at the predefined times, diluted with 18 mL filtered water and further used to determine the release of silver by ICP–MS.

Calibration standards were prepared by serial dilution of a certified multi-element standard solution in ultrapure water (18 MΩ·cm, Milli-Q^®^, Millipore, Bedford, MA, USA) to concentrations of 0, 1, 5, 10, 25, 50, and 100 μg·L^−1^ (ppb). The calibration curve for silver determination was generated within this concentration range. The analysis samples, originally in aqueous media, were acidified before measurement by adding 50 μL of concentrated nitric acid (HNO_3_, approximately 65%) to each sample to stabilize the analyte and prevent adsorption losses. No further dilution of the samples was carried out. Measurements were performed using an Agilent 8800 Triple Quadrupole ICP–MS system (Agilent Technologies, Tokyo, Japan), following the instrument-tuning procedures recommended by the manufacturer. The system is equipped with two quadrupoles separated by a helium-filled collision/reaction cell (CRC), which helps to reduce spectral interferences. Silver was detected using its isotope, ^107^Ag. The operating parameters included: RF power of 1150 W, sample insertion depth of 8.0 mm, carrier gas flow rate of 1.00 L/min, nebulizer pump speed of 0.10 rps, spray chamber temperature maintained at 2 °C, and helium cell gas flow of 7 mL/min. A dwell time of 10 ms was used for silver detection. During analysis, nickel cones were employed.

#### 4.5.9. Evaluation of Bacterial Viability and Metabolic Activity

The antibacterial activity of hybrid hydrogels was evaluated by assessing the viability of the *S. aureus* strain (ATCC strain 29213) after 24 h in contact with the experimental materials.

The bacterial strain was cultured from an isolated colony on TSA agar into TSB and was incubated at 37 °C for 16–18 h. The inoculum was adjusted to an optical density corresponding to the 0.5 McFarland standard (~1–2 × 10^8^ CFU/mL). It was diluted in a fresh medium until the 10^6^ CFU/mL was achieved for the exposure assays.

Hybrid materials were placed in the sterile wells and the inoculum was added, followed by incubation for 24 h at 37 °C. Parallel samples were prepared for each material, alongside bacterial (without material) and sterility controls. Subsequently, the bacterial suspensions were homogenized by repeated pipetting; ten-fold serial dilutions were performed in PBS. The 100 µL aliquots were plated onto TSA using the spread plate technique. The plates were then incubated for 18–24 h at 37 °C and colonies were counted from plates. The total number of colony-forming units was calculated using Equation (5):

(5) $$CFU/mL=\frac{N\;\times \;D}{V}$$

where *N*—number of colonies, *D*—dilution factor, *V*—plated volume (0.1 mL). The percentage of viability was expressed by normalizing the CFU/mL value of each material to the untreated control, considered as 100%. Results were expressed as mean ± standard deviation for a minimum of three experimental replicates.

The XTT assay was performed to quantify the metabolic activity of *S. aureus* following exposure to the hybrid hydrogels. The bacterial suspension was obtained as previously stated. Hydrogels were placed in sterile plates and 1 mL of bacterial suspension was added. A positive and a negative control were also added to the plate. After incubation at 37 °C for 24 h, the suspensions were transferred to 96-well microplates. XTT was prepared according to the manufacturer’s instructions by dissolving the salts and adding menadione solution as an activator. For every 100 µL of bacterial suspension, 50 µL of XTT solution was added, followed by 1–3 h of incubation protected from light. The reduction of XTT to formazan product was quantified by measuring the absorbance at 450 nm using a spectrophotometer (SkanIT, Thermo Scientific). Additionally, the hydrogels were tested against 4 more strains in the same conditions (*S. epidermidis*, *MRSA*, *P. aeruginosa*, *E. coli*). Absorbance values were measured in triplicate for each sample.

#### 4.5.10. Biocompatibility

Cell viability and metabolic activity were evaluated using the MTT Cell Proliferation Assay Kit (Roche Diagnostics), following the manufacturer’s instructions. Human dermal fibroblasts (HDF) were seeded at a density of 1 × 10^4^ cells/well in 24-well plates and cultured in DMEM supplemented with 10% fetal bovine serum (FBS) at 37 °C in a humidified atmosphere with 5% CO_2_. After 24 h, the culture medium was replaced and the cells were exposed to the tested hydrogels.

Following 24 h incubation, the culture medium was removed and the samples were washed with PBS. Subsequently, MTT reagent (final concentration: 0.5 mg/mL) was added to each well and incubated for 4 h at 37 °C. The resulting formazan crystals were solubilized using the provided solubilization solution; the absorbance was measured at 550 nm using a microplate reader (Multiskan FC, Thermo Scientific). The absorbance values were directly proportional to the number of metabolically active cells.

Cytotoxicity was assessed using the Lactate Dehydrogenase (LDH) Cytotoxicity Detection Kit (Roche Diagnostics), according to the manufacturer’s protocol. After 24 h exposure of HDF cells to the hydrogels, 50 µL of cell culture supernatant from each well was transferred to a 96-well plate.

A total of 100 µL of reaction mixture (prepared according to the kit instructions) was added to each well and incubated for 15–20 min at room temperature in the dark. The enzymatic reaction results in the formation of a colored product proportional to LDH release, which reflects membrane damage and cell death. The absorbance was measured at 490 nm, with a reference wavelength of 600 nm, using a microplate reader (Tecan Infinite 200 Pro, GENios Tecan, Ramsey, MN, USA). Results were expressed as OD values.

Cell viability and morphology were further assessed using the Live/Dead Viability/Cytotoxicity Kit (Invitrogen, Thermo Fisher Scientific, Waltham, Massachusetts, USA). HDF cells were seeded at a density of 1 × 10^4^ cells/well and cultured in the presence of hydrogels for 24–72 h under standard conditions (37 °C, 5% CO_2_).

After incubation, the culture medium was removed and the samples were gently washed with PBS. A staining solution containing calcein-AM (for live cells, green fluorescence) and ethidium homodimer-1 (for dead cells, red fluorescence) was prepared according to the manufacturer’s instructions and added to each well. The samples were incubated for 30–60 min in the dark at room temperature.

Fluorescence images were acquired using a Zeiss Axioscope fluorescence microscope (AxioCam, Oberkochen, Germany) to evaluate cell viability, morphology, and attachment on the hydrogel surfaces.

### 4.6. Statistical Significance

Measurements were performed in triplicate (*n* = 3) unless otherwise mentioned. The results are expressed as mean ± standard deviation (SD). Statistical relevance was assessed using one-way ANOVA method using the mathematical model Holm–Bonferroni in software OriginLab 2009. Significant statistical differences were considered for *p* < 0.05.

## Figures and Tables

**Table 1:** Parameters of the diffusion.

	PAAm	PTA90-10	PTA80-20	PTA70-30	PTA60-40	PTA50-50
k	0.00558	0.00996	0.00851	0.00855	0.00642	0.0057
n	0.6212	0.5025	0.5522	0.5488	0.5822	0.7282
R^2^	0.994	0.984	0.999	0.996	0.998	0.982

**Table 2:** Summarized results obtained from uniaxial compression (Young’s modulus—E), mechanical studies at the microscale and contact angle of hybrid hydrogels and pristine PAAm.

	PAAm	PTA90-10	PTA80-20	PTA70-30	PTA60-40	PTA50-50
E (kPa)	80.23 ± 1.15	60.08 ± 0.74	51.64 ± 0.58	32.70 ± 1.25	26.2 ± 0.85	18.76 ± 0.5
G′ (kPa)	9.72 ± 0.41	7.57 ± 0.4	10.34 ± 0.17	6.68 ± 0.35	2.53 ± 0.16	0.98 ± 0.13
G″ (kPa)	0.28 ± 0.027	0.26 ± 0.008	0.11 ± 0.005	0.04 ± 0.005	0.15 ± 0.007	0.13 ± 0.01

**Table 3:** Formulation denomination and compositional details.

Denomination	PAAm Precursor Solution (v/v%)	TOCNF–AgNPs (v/v%)
PAAm	100	-
PTA90-10	90	10
PTA80-20	80	20
PTA70-30	70	30
PTA60-40	60	40
PTA50-50	50	50

## Data Availability

The original contributions presented in the study are included in the article. Further inquiries can be directed at the corresponding author.

## Supplementary Materials

The following supporting information can be downloaded from https://www.mdpi.com/article/10.3390/gels12060457/s1. Figure S1. G′ and G″ as a function of strain, for all samples; Figure S2. G′ and G″ variation with frequency in the interval 0.1–100 Hz; Figure S3. G′ and G″ variation depending on alternating strain in consecutive cycles; Table S1. Data were obtained from TGA analysis using the software Proteus.
