# Supplementary material for: AgNPs–Cellulose Nanofiber/Polyacrylamide Hydrogels as an Antibacterial Platform for Soft Tissue
Source: Gels. 2026 May 23;12(6):457. doi: 10.3390/gels12060457 (PMC13297931; doi:10.3390/gels12060457)
Supplement: Supplementary file 1 [file gels-12-00457-s001.zip › gels-4225458-supplementary.pdf]

AgNPs-cellulose nanofiber/polyacrylamide hydrogels as an antibacterial platform for soft tissue

Ioana Maria Marinescu [1], Andrada Serafim [2], Elena Olaret [2], Bogdan Stefan Vasile [3,4], Mona Mihailescu [5], Gratiela Gradisteanu Pircalabioru [6], Kristin Syverud [7], Stian Kreken Almeland [8], Samih Mohamed-Ahmed [9], Kamal Mustafa [9], Esko Kankuri [10], Cristian Botezatu [11,12], Bogdan-Stelian Mastalier-Manolescu [11,12], Alexandra Catalina Birca [13] and Izabela-Cristina Stancu [1,14,*]

[1]Advanced Polymer Materials Group, Faculty of Chemical Engineering and Biotechnologies, National University of Science and Technology POLITEHNICA Bucharest, 011061 Bucharest, Romania;
[2]Advanced Polymer Materials Group, CAMPUS Research Institute, National University of Science and Technology POLITEHNICA Bucharest, 060042 Bucharest, Romania;
[3]Research Center for Advanced Materials, Products and Processes, National University of Science and Technology POLITEHNICA Bucharest, 060042 Bucharest, Romania;
[4]National Research Center for Micro and Nanomaterials, Faculty of Chemical Engineering and Biotechnologies, National University of Science and Technology POLITEHNICA Bucharest, 060042 Bucharest, Romania;
[5]Holographic Imaging and Processing Laboratory, Physics Department, National University for Science and Technology POLITEHNICA of Bucharest, 060042 Bucharest, Romania;
[6]Faculty of Biology, University of Bucharest, 050097 Bucharest, Romania;
[7]RISE PFI, Department of Chemical Engineering, Norwegian University of Science and Technology (NTNU), NO-7034 Trondheim, Norway;
[8]Department of Plastic, Hand and Reconstructive Surgery, Norwegian National Burn Center, Haukeland University Hospital, Bergen, Norway;
[9]Center of Translational Oral Research (TOR), Department of Clinical Dentistry, University of Bergen, Bergen, Norway;
[10]Faculty of Medicine, University of Helsinki, Helsinki, Finland;
[11]Surgery Department, University of Medicine and Pharmacy Carol Davila,050474 Bucharest, Romania;
[12]Surgery Department, Colentina Clinical Hospital, 020125 Bucharest, Romania
[13]Department of Science and Engineering of Oxide Materials and Nanomaterials, National University of Science and Technology POLITEHNICA Bucharest, 011061 Bucharest, Romania;
[14]Faculty of Medical Engineering, National University of Science and Technology POLITEHNICA Bucharest, 011061 Bucharest, Romania
*Correspondence: izabela.stancu@upb.ro

Supporting information

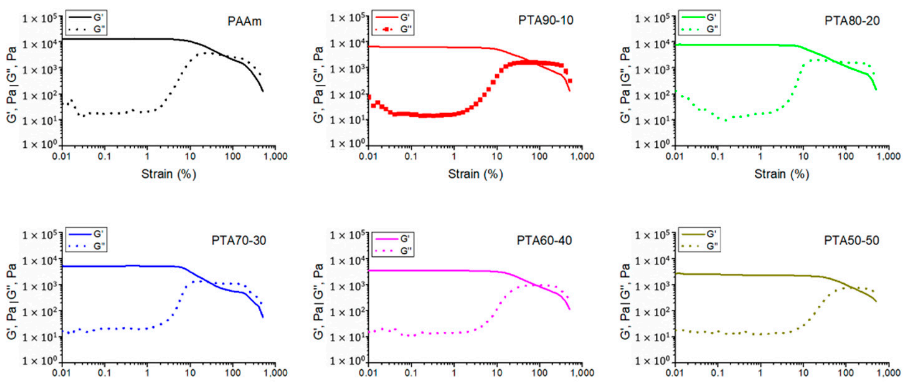

Figure S1. G’ and G” as a function of strain, for all samples.

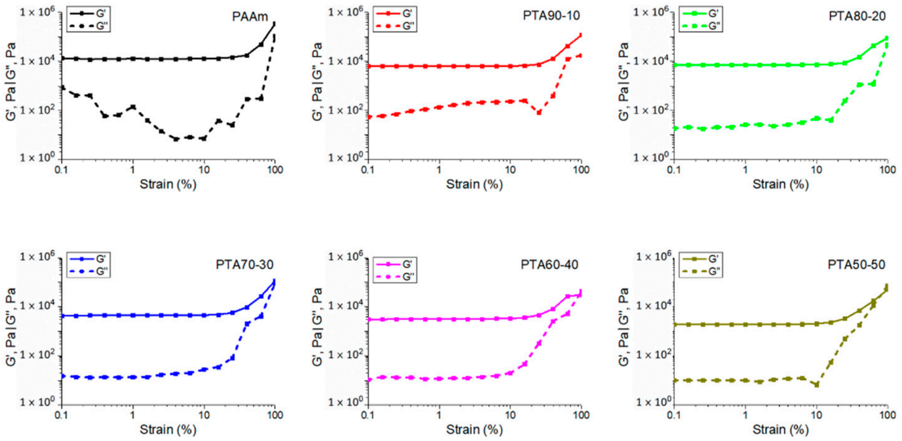

Figure S2. G’ and G” variation with frequency in the interval 0.1 – 100 Hz.

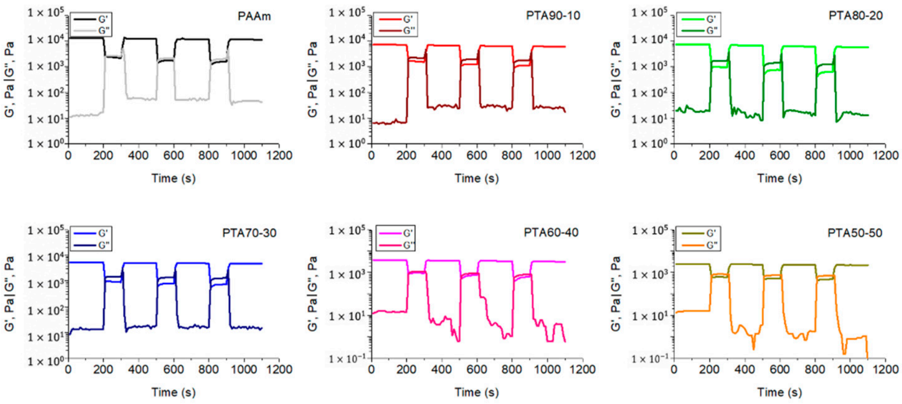

Figure S3. G’ and G” variation depending on alternating strain in consecutive cycles.

Table S1. Data obtained from TGA analysis using the software Proteus.

Sample	T3% (°C)	Residual mass (%)	Peaks (°C)		
PAAm	155.1	2.57	209.5		385
PTA90-10	176.5	16.11	182.7	305.3	389.9
PTA80-20	164.3	20.49	182.9	293	391
PTA70-30	163.5	21.49	179.2	284.6	384.1
PTA60-40	135.4	21.81	208.6	283.8	376.8
PTA50-50	139.8	23.99	219.8	288.9	382.6
TOCNF	112.3	27.85	236.9	306	
TOCNF-AgNPs	125	43.43	220.7	241.8	
